# Luminescence Nanomaterials and Applications

**DOI:** 10.3390/nano13061047

**Published:** 2023-03-14

**Authors:** Wei Chen, Derong Cao

**Affiliations:** 1 Department of Physics, The University of Texas at Arlington, Arlington, TX 76019-0059, USA; 2 Department of Chemistry, South China University of Technology, Guangzhou 510641, China

## 1. Introduction

We are pleased to introduce to you this Special Issue of *Nanomaterials* on ‘Luminescence Nanomaterials and Applications’. Luminescence is a phenomenon that we experience daily in our work and lives. Emerging nanotechnology and quantum dots offer a new class of materials to make our lives better. For example, the applications of luminescent nanoparticles for medical research are taking advantage of their high quantum yield, multi-colors, high photostability, large surface-to-volume ratio, surface functionality, and small size. For solid-state displays, they can provide more colors by simply adjusting the size of the particles. In this Special Issue, we discuss nanomaterials with quantum size confinement, photoluminescence, upconversion, thermoluminescence, and long persistence, as well as their potential applications in cell labeling, imaging, detection, and sensing. This Special Issue also covers the synthesis of luminescence nanomaterials, applications for in vitro and in vivo imaging, detection based on fluorescence resonance energy, and the applications of luminescent nanoparticles for photodynamic activation and solid-state displays, as well as new materials and structures, such as perovskite quantum dots, and novel phenomena, such as aggregation-induced emissions. In total, we have 15 papers (2 reviews and 13 research articles) for this Special Issue, which are summarized below:

## 2. Upconversion Luminescence Nanomaterials

Upconversion nanomaterials can emit high-energy lights when excited with two or more low-energy photons. They can produce ultraviolet (UV)-visible or near-infrared (NIR) light upon excitation with NIR light, depending on size or dopants, owing to their unique properties, such as good optical stability, narrow emission band, large anti-Stokes spectral shift, high levels of light penetration in biological tissues, long luminescence lifetime, and high signal-to-noise ratio. The review paper by Dr. Jigang Wang and his collaborators systematically introduced the physical mechanism of upconversion luminescence nanomaterials and their potential applications in bioimaging, detection, photodynamic therapy, and therapeutics [1].

Upconversion nanocrystals converting near-infrared light into high-energy UV emissions may provide many exciting opportunities for drug release, photocatalysis, photodynamic therapy, and solid-state lasing. However, a key challenge is their low conversion efficiency. For that, Dr. Chuanyu Hu’s team [2] proposed and developed dye-sensitized and heterogeneous core–shell lanthanide nanostructures for ultraviolet upconversion improvement. They systematically investigated the main factors on ultraviolet upconversion emission. Interestingly, they found a method for a largely promoted multiphoton upconversion, which provides more opportunities for applications in biomedicine, photo-catalysis, and environmental science.

## 3. Luminescence for Solid-State Lighting, Displays, and Anti-Counterfeiting

White-light-emitting diodes show great promise for replacing traditional lighting devices because of their high efficiency, low energy consumption, and long lifetime. Metal organic frameworks (MOFs) are good materials for white-light emissions. The encapsulation of organic dyes is a simple way to obtain luminescent MOFs. In a review, Dr. Derong Cao and his collaboration team summarized the recent research on the design and construction of dye-encapsulated MOFs phosphors and their potential applications [3].

Dr. Dangli Gao and her collaboration team reported the optical properties of Zn_3_Ga_2_GeO_8_:Mn phosphors that could be modified by different preparation methods, including a hydrothermal method and solid-state diffusion combined with a non-equivalent ion-doping strategy [4]. Consequently, Mn-doped Zn_3_Ga_2_GeO_8_ phosphors prepared by a hydrothermal method showed an enhanced red emission at 701 nm and a green persistent luminescence at 540 nm, while the phosphors prepared by solid-state diffusion in combination with hetero-valent doping only exhibited an enhancement in the single-band red emission. Furthermore, the substitution of hetero-valent dopant ion Li^+^ into different sites can change the emission colors. These fantastic phenomena were discussed in detail in the paper [4].

The study of room-temperature phosphorescent carbon quantum dots is important for various applications. Dr. Hongmei Yu et al. [5] successfully fabricated matrix-free carbonized polymer dots (CPDs) that can produce green room-temperature phosphorescence under dual-mode visible- and ultraviolet-light excitations. Hydrogen bonding can provide a space protection and stably excite the triplet state. This self-matrix structure effectively avoids the non-radiative transition by blocking the intramolecular motion of CPDs. The long lasting room-temperature phosphorescence is good for applications in anti-counterfeiting.

## 4. Particle Based Sensing Technology

Early cancer detection is important, and plenty of sensors are being explored. Dr. Liu and her collaboration team reported novel lanthanide-upconverted NaYF_4_:Yb,Tm fluorescence probes, which can detect cancer-related specific miRNAs in very low concentrations [6]. The detection is based on emissions at 345, 646, and 802 nm upon excitation at 980 nm. The two common proteins, miRNA-155 and miRNA-150, were captured by the designed fluorescent probes. The probes can effectively distinguish miRNA-155 from partial- and complete-base mismatched miRNA-155, which is critical for early cancer detection.

Sulfur quantum dots (SQDs) are considered potential green nanomaterials because they have no heavy metals. Dr. Yang [7] and his collaboration team prepared SQDs by a microwave-assisted method using sulfur powder as a precursor. SQDs show the highest emission at 470 nm when excited at 380 nm and have a good sensitivity and selectivity in alkaline phosphatase detection.

Radiation detection and dosimetry are old questions but pose new challenges for security and safety. As such, Dr. Abd Khamim Ismail et al. [8] examined the thermoluminescence dosimetry behaviors of Ag-ZnO films. The dose–response revealed high linearity up to 4 Gy. The proposed sensitivity was 1.8 times higher than the TLD 100 dosimeters.

## 5. Particle-Based Therapeutics

Chemodynamic therapy (CDT) has received extensive research attention in recent years. However, the efficiency of CDT is influenced by H_2_O_2_ limitations in the tumor. In this issue, Dr. Yang Shu and her collaborators [9] described a novel core–shell nanostructure, namely a Cu-metal organic framework (Cu-MOF)/glucose oxidase (GOD)@hyaluronic acid (HA) (Cu-MOF/GOD@HA), for the purpose of improving CDT efficacy by increasing H_2_O_2_ concentration and cancer cell targeting. The CDT enhancement as a result of GOD and HA effects in Cu-MOF/GOD@HA was confirmed for both in vitro cell and in vivo animal studies.

Photothermal therapy has been widely tested in treating bacterial infections [10,11]. Weiwei Zhang et al. [12] tested the growth inhibition of Staphylococcus aureus by using a very low concentration of vancomycin and applying photothermal therapy with MoS_2_. MoS_2_-Van-FITC with near-infrared irradiation significantly inhibited S. aureus growth, reaching an inhibition rate of 94.5%, indicating its possible use as a wound-healing agent.

Dr. Oleg A. Yeshchenko et al. [13] presented the thermoresponsive Zinc-TetraPhenylPorphyrin-based hybrid nanosystem. The shrinking of D-g-PNIPAM macromolecules during a thermally induced phase transition leads to the release of both ZnTPP molecules and Au NPs from the ZnTPP/D-g-PNIPAM/AuNPs macromolecule. The three-fold enhancement of singlet oxygen production with surface plasmon resonance is critical for clinic applications.

## 6. New Materials and Structures

Bovine-serum-albumin-embedded Au nanoclusters are thoroughly investigated by Radek Ostruszka [14] using continuous-wave electron paramagnetic resonance, light-induced EPR, etc. In addition to the presence of Au(0) and Au(I) oxidation states in BSA-AuNCs, a significant amount of Au(II) was detected, which may come from a disproportionation event occurring within NCs: 2Au(I) − Au(II) + Au(0).

Haibin Li et al. [15] reported on n-p Bi_2_O_2_CO_3_/α-Bi_2_O_3_ heterojunction microtubes and studied their photocatalytic activities under visible-light irradiation. The results indicated that Bi_2_O_2_CO_3_/α-Bi_2_O_3_ with a Bi_2_O_2_CO_3_ mass fraction of 6.1% exhibited higher photocatalytic activity than α-Bi_2_O_3_.

Obtaining drinking water from seawater has always been a long-term goal. Here, Dr. Zhongxin Liu et al. [16] reported on the use of graphene-loaded nonwoven fabric membranes coated with graphene oxide for seawater purification. The photothermal membrane is expected to be suitable for regional water purification and seawater desalination due to its high light absorption, strong heating effect, and its evaporation rate, which is about five times higher than that of non-woven fabric.

In this issue Dr. Yiqun Li et al. [17] synthesized a novel carboxymethylcellulose–polyaniline-film-supported copper catalyst (CuII/I@CMC-PANI) and used it as a dip catalyst for aldehyde–alkyne–amine coupling reactions with a high yield of 97%. They found that CuII/I@CMC-PANI, as a good dip catalyst, is very useful in organic synthesis due to its easy fabrication, convenient deployment, superior catalytic activity, and high reusability.

We would like to thank the *Nanomaterials* Editorial Office for the opportunity to edit this Special Issue, as well as all the authors for their valuable contributions and reviewers for their valuable comments. This Special Issue would not have been possible without them. We hope that this Special Issue can offer some valuable information and guidance for future research directions.
